# Health Assessments of Koalas after Wildfire: A Temporal Comparison of Rehabilitated and Non-Rescued Resident Individuals

**DOI:** 10.3390/ani13182863

**Published:** 2023-09-09

**Authors:** Murraya R. Lane, Arianne Lowe, Jelena Vukcevic, Robert G. Clark, George Madani, Damien P. Higgins, Luke Silver, Katherine Belov, Carolyn J. Hogg, Karen J. Marsh

**Affiliations:** 1 Research School of Biology, The Australian National University, Canberra, ACT 2601, Australia; karen.marsh@anu.edu.au; 2 Stromlo Veterinary Services, P.O. Box 3963, Weston, ACT 2611, Australia; lowearianne@outlook.com; 3 The Foreshore Vet, Kingston, ACT 2604, Australia; jvuk0156@gmail.com; 4 Research School of Finance, Actuarial Studies and Statistics, The Australian National University, Canberra, ACT 2601, Australia; robert.clark@anu.edu.au; 5 School of Environmental and Life Sciences, The University of Newcastle, Callaghan, NSW 2308, Australia; chiro_ptera@hotmail.com; 6 Sydney School of Veterinary Science, The University of Sydney, Sydney, NSW 2006, Australia; damien.higgins@sydney.edu.au; 7 School of Life and Environmental Sciences, The University of Sydney, Sydney, NSW 2006, Australia; luke.silver@sydney.edu.au (L.S.); kathy.belov@sydney.edu.au (K.B.); carolyn.hogg@sydney.edu.au (C.J.H.)

**Keywords:** marsupial folivore, bushfires, health parameters, rehabilitation, body condition

## Abstract

**Simple Summary:**

Bushfires are a regular occurrence in the Australian landscape. The 2019/20 megafires were unprecedented in intensity and scale, impacting many native species. Koalas were particularly impacted by the fires, with many of those coming into care requiring extensive rehabilitation and treatment. Very little is known about the health parameters of rehabilitated koalas following their release and how these parameters may differ between individuals living in a burnt landscape compared to those in an unburnt landscape. This study tested haematological and serum biochemical parameters, chlamydial shedding and body condition scores of rehabilitated koalas and non-rescued residents living in burnt and unburnt habitats. All koalas received a health check 5–9 months post-fire and one 12–16 months post-fire. Rehabilitated koalas also received a health check when coming into care initially. While some variation in parameters was recorded, the majority of measurements in each koala group fell within the normal reference ranges, suggesting that resident and rehabilitated koalas were in good health at the time of release and when recaptured. These results show that koalas can be supported in burnt landscapes (provided there is adequate nutrition) and that the health of rehabilitated koalas is not compromised by returning them to burnt habitats.

**Abstract:**

Many koalas (*Phascolarctos cinereus*) required rehabilitation after the 2019/20 Australian megafires. Little is known about how the post-release health of rehabilitated koalas compares to non-rescued resident koalas. We evaluated health parameters in rehabilitated koalas and resident koalas in burnt and unburnt habitat in southern New South Wales, Australia. Health checks were undertaken within six weeks of fire (rehabilitated group), 5–9 months post-fire and 12–16 months post-fire. Body condition improved significantly over time in rehabilitated koalas, with similar condition between all groups at 12–16 months. Rehabilitated koalas therefore gained body condition at similar rates to koalas who remained and survived in the wild. The prevalence of *Chlamydia pecorum* was also similar between groups and timepoints, suggesting wildfire and rehabilitation did not exacerbate disease in this population. While there was some variation in measured serum biochemistry and haematology parameters between groups and timepoints, most were within normal reference ranges. Our findings show that koalas were generally healthy at the time of release and when recaptured nine months later. Landscapes in the Monaro region exhibiting a mosaic of burn severity can support koalas, and rehabilitated koala health is not compromised by returning them to burnt habitats 4–6 months post-fire.

## 1. Introduction

Fire is a natural and often essential part of the Australian landscape, with the dry sclerophyll forests in the south-east region considered to be one of the most fire-prone regions in the world [1,2,3]. Australia has experienced many wildfires throughout history; however, the 2019/20 bushfire season was unprecedented in both severity and scale [4]. Between August 2019 and March 2020, 12.6 million hectares burnt on the eastern side of Australia across the states of Victoria, New South Wales (NSW), Queensland and South Australia [4,5]. The effects on native wildlife were significant, with losses estimated in the billions [6].

The koala (*Phascolarctus cinereus*) accounted for about 75% of the wildlife brought into emergency triage units in Victoria [7]. Hundreds of koalas were also rescued in NSW [8] and South Australia [9], with rescue efforts continuing for weeks to months after the fires. There is no uniform approach to releasing fire-affected koalas after rehabilitation; some are returned to their rescue locations in burnt habitats while others are translocated to nearby unburnt habitats [7,8,9]. Both approaches have risks that have not been adequately quantified. For example, translocated koalas often move further from their release sites relative to koalas that remain in situ, which can place them at greater risk of predation and vehicle strikes [10,11]. In contrast, burnt habitats may be lower quality with less food available and increased exposure to predators [12,13].

Koalas are dependent on *Eucalyptus* trees for both food and shelter [14,15], and these resources are often depleted or patchily distributed in burnt areas [16]. Many eucalypt species can regenerate from epicormic buds on branches and stems relatively quickly after a fire [17,18,19,20,21]. However, it is unclear how these changes affect habitat quality for koalas, and hence whether fire influences the longer-term health of individuals and populations living in burnt habitats. For example, in some unburnt areas, diseases such as chlamydiosis and, potentially, koala retrovirus (KoRV) can have substantial impacts on animal health, breeding success and population density [22]. Chlamydial infertility in particular is considered to play a major role in the decline in some koala populations [23,24]. Environmental stressors, such as bushfires, have been hypothesised to further exacerbate disease, as well as to influence susceptibility to infections in wildlife in general [25]. Given the extent of the 2019/20 megafires (e.g., more than 3.5 million hectares (or 25%) of suitable koala habitat burnt in NSW [5]) and that fires are predicted to become more common and extreme with climate change [26], there is a critical need to understand whether there are longer-term health risks for rehabilitated koalas released into burnt habitats and whether the health of koalas in burnt habitats differs from those in unburnt habitats.

During and following the 2019/20 megafires, community members and volunteers rescued 33 koalas from fire-impacted areas in the NSW Snowy Monaro Shire [8], of which 31 had initial health data available for this study. Koalas that recovered after being in care were predominantly released at their rescue location into a burnt habitat. A third of these koalas were also fitted with GPS tracking collars as part of a post-release monitoring program, along with 25 non-rescued resident koalas from the same vicinity. The resident koalas occupied a mosaic of burnt and unburnt habitats, providing a unique opportunity to compare the health of rehabilitated koalas before and after release with their non-rescued counterparts. Where possible, we collected data on body condition, chlamydial shedding and blood biochemistry and haematology at three timepoints for rehabilitated koalas: the time of rescue (January to March 2020), the time of release (June to December 2020) and the time of collar removal (February to June 2021). We collected the same data for resident koalas during the latter two time periods.

The comparative health data collected in this study provide a novel opportunity to address a key gap in our understanding of whether rehabilitated koalas should be returned to burnt habitats after their recovery. Furthermore, the study provides important baseline information about the general health and background levels of disease in a significant but understudied koala population, as well as improving the knowledge base about disease prevalence and severity across the distribution of the koala [22,27]. Many koala populations across south-eastern Australia are in decline due to a combination of habitat destruction, fragmentation, wildfire, vehicle strikes, dog attacks and disease [12,22,28,29]. Accordingly, koalas are federally listed as ‘Endangered’ in Queensland, NSW, and the Australian Capital Territory [30]. This data will also provide important information about the management implications of rescuing and rehabilitating koalas after fire.

We expected that rehabilitated koalas would be in poorer body condition when they were first rescued but would recover to a similar or possibly better condition at their time of release compared to resident koalas living in a fire-impacted landscape. We also anticipated that koalas in a burnt habitat (rehabilitated or resident) would be in poorer body condition than koalas in an unburnt habitat if food quantity or quality were limiting. If fire or rehabilitation affected koala condition, we also expected to see indicators of poorer health in blood biochemistry and haematology parameters. Finally, we expected that there may be differences in the prevalence or the severity of symptoms of chlamydial disease between koalas in burnt and unburnt habitats.

## 2. Materials and Methods

### 2.1. Study Area

The Snowy Monaro Shire in NSW, Australia, incorporates the sub-alpine region that extends from the Australian Capital Territory in the north to the Victorian border in the south, west to the Kosciuszko Ranges and east to the Kybeyan range. Within the Snowy Monaro Shire, there are three main bioregions, including the South Eastern Highlands (59%), Australian Alps (23%) and the South East Corner Region (18%) [31]. Koalas are known to occur in the Macanally–Numeralla Ranges, east of the Monaro tableland, within the South Eastern Highlands bioregion [32]. These areas are comprised of north–south ridges with steep sloping terrain and an elevational range of 800–1233 m. The main vegetation class in the area is Southern Tablelands dry sclerophyll forest, which is dominated by red stringybark (*Eucalyptus macrorhyncha)* and scribbly gum (*E. rossii*) [13]. Brittle gum (*E. mannifera)* and broad-leaved peppermint (*E. dives)* are also abundant in this area [33]. Ribbon gum (*E. viminalis*) and candlebark (*E. rubida)* are present in creek lines, and snow gums (*E. pauciflora)* are found on slopes at higher elevations [34,35]. Understory vegetation is dominated by *Acacia* spp. [36].

### 2.2. Koala Groups

**Figure 1:**
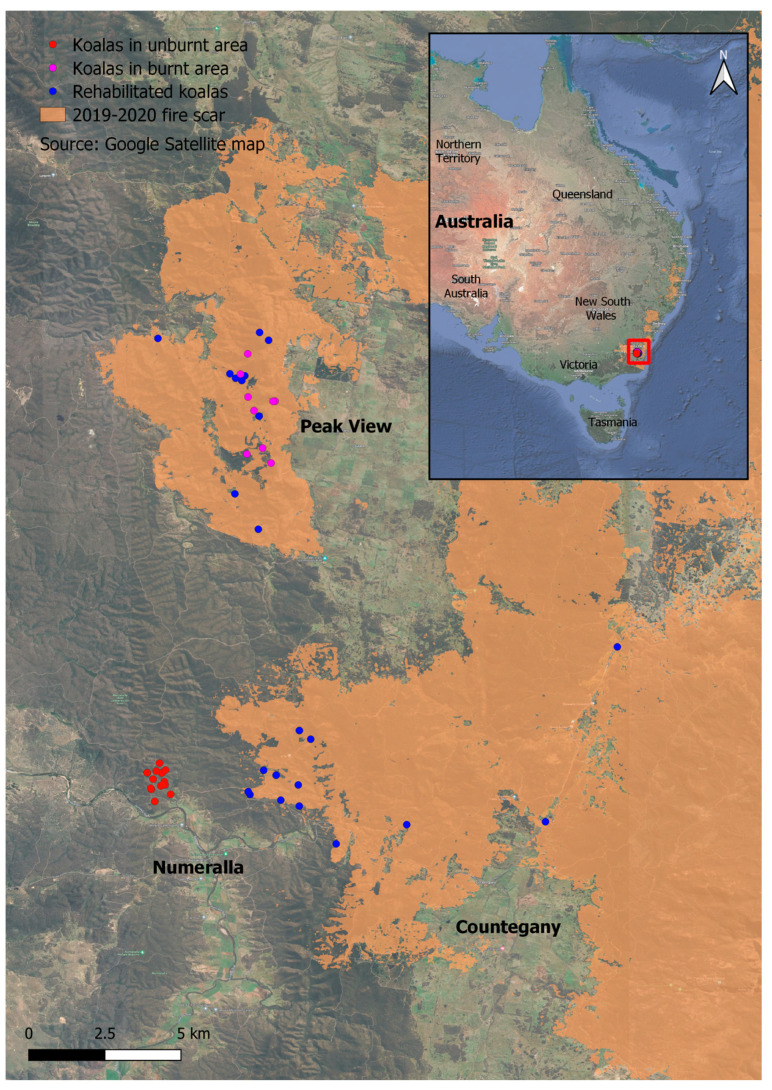
Capture locations of all rescued koalas (blue) as well as resident koalas captured in burnt habitat (pink) or unburnt habitat (red) against the 2019–2020 fire scar (orange) [39]. The inset shows the location of the study site in south-eastern Australia.

Following bushfires in late January 2020, we obtained health data from 31 koalas (15 males, 16 females) that were rescued from Peak View (36.07° S, 149.38° E), Numeralla (36.17° S, 149.33° E) and Countegany (36.18° S, 149.45° E) in the Snowy Monaro Shire (Figure 1). After veterinary triage (described in more detail in the “data collection” section) and initial care at the Australian National University, the koalas were transported to licensed local wildlife carers, where their care continued until release. Twelve of these koalas were also selected for post-release GPS monitoring (the “rehabilitated” group; nine males and three females). Release of these koalas began in June 2020, with all koalas being released at their rescue locations by December 2020.

Between May and September 2020, additional non-rescued resident koalas were located and captured in Peak View (*n* = 9) and Numeralla (*n* = 16). Koalas were located either by daylight ground-based searches, or by a drone fitted with a thermal imaging camera [37]. They were captured using the noose and flag method described in work by Madani et al. [38] and, after being examined by an experienced wildlife veterinarian (see “data collection” section) and fitted with a LiteTrack 60 VHF/GPS tracking collar (Lotek, Havelock, New Zealand), were released on the same day. The koalas captured at Numeralla were found in unburnt habitat, approximately 4 km from the nearest fire ground (the “unburnt resident” group; 5 males and 11 females), while those captured in Peak View were in a habitat within the burn scar (the “burnt resident” group; 4 males and 5 females) (Figure 1). All koalas who retained their collar were recaptured eight to nine months later for collar removal and a final veterinary check (see “data collection” section). The Supplementary Material (Table S1) contains a full list of koalas assessed at each point in the study.

### 2.3. Data Collection

Health data were collected from each koala at up to three different timepoints depending on their group (Table 1). These timepoints were:

- Rescue: an initial health check within 24 h of rescue for the rehabilitated group only.
- Pre-release: a pre-release check (5–9 months after rescue) for rehabilitated koalas who were selected for post-release monitoring, and a pre-collaring check for burnt resident and unburnt resident koalas captured for the monitoring study.
- Recapture: a final check up to nine months after the pre-release check for all monitored koalas who retained their tracking collars.

Health checks were conducted by wildlife veterinarians under heavy sedation or general anaesthesia (see Appendix A (A1) for Koala Health Hub’s koala health protocol for sampling of koalas following capture and restraint). Premedication with Alfaxalone (Jurox 10 mg/mL; up to 3 mg/kg intramuscular) was followed by maintenance on oxygen delivered by a face mask and/or isoflurane (VCA 1 mL/mL at 1–3% to effect) with an oxygen flow of 2 L/min. Koalas were maintained in lateral recumbency, with spontaneous breathing and relaxed muscle tone for the physical examination and data collection. Anaesthesia was monitored with heart rate, respiratory rate and temperature taken at regular intervals and depth assessed via palpebral and withdrawal reflexes. Koalas were checked for the presence of burns or any other clinically important findings, and a variety of general parameters were recorded, including sex, presence of pouch young (for females), age (from tooth wear score) and weight. Tooth wear score was determined by assessing the wear of the fourth upper premolars according to [40]. Veterinarians also assessed physical condition, looking at abdominal fill status and aural health. Body condition was assessed by one of two wildlife veterinarians using a standardised technique of palpation of the muscles on either side of the protruding ridge of bone on the scapula (spine of the scapula), and was scored out of 5 [9,41,42,43].

Blood was collected from the cephalic vein into serum and EDTA tubes for haematology, biochemistry and genetic analysis, and blood smears were made in the field. During cold conditions, blood draw was assisted by wrapping the forelimb in a warm towel. Ocular swabs were collected by gently and carefully rolling the swab (premoistened with sterile saline) across conjunctiva on both eyes, while urogenital swabs were collected from the urogenital sinus (females) or penile urethra (males). In the first health check only (rescue or pre-release check depending on koala group), an ear tissue biopsy was collected in 80% ethanol, a microchip (Trovan, UK) placed subcutaneously within the intrascapular space and an individually numbered plastic ear tag (3.5 cm × 1 cm) placed into the right pinna of females and left pinna for males, using an aseptic technique. At the pre-release check, koalas were also fitted with a LiteTrack 60 VHF/GPS tracking collar (Lotek, Havelock, New Zealand) that weighed 80 g around the neck, with at least two finger widths of space between the neck and collar. Each collar had a unique frequency that was tested prior to releasing the koala. An elastic weak link was incorporated into each collar to ensure release in the event of snagging.

### 2.4. Sample Processing

After collection, blood and swab samples were transported to the Australian National University. Upon arrival, 0.5 mL blood in EDTA was pipetted into a new vial for haematological analysis. Serum tubes were centrifuged at 6500× *g* for 10 min and the serum was transferred to a new vial for biochemical analysis. Blood and serum samples were refrigerated at 4 °C for up to 48 h and swabs were stored at ambient temperature for up to five days, pending analysis at the Sydney School of Veterinary Science, University of Sydney. Haematological parameters measured included PCV (packed cell volume), TPP (total protein), haemoglobin, erythrocytes, MCV (mean corpuscular volume), MCHC (mean corpuscular haemoglobin concentrations), MCH (mean corpuscular haemoglobin), platelet count, leukocytes, neutrophils, lymphocytes, neutrophil/lymphocyte ratio, monocytes, eosinophils and basophils. Haematocrit and total plasma protein were determined by capillary tube centrifugation and refractometer, respectively. White cell count was determined using a Sysmex haematological analyser (XN-1000RF Vet, Roche Diagnostics, Basel, Switzerland) and differential counts obtained by light microscopy.

Biochemical parameters measured included urea, creatinine, AST (aspartate aminotransferase), ALP (alkaline phosphatase), GGT (gamma-glutamyl transferase), bilirubin, phosphate and ALT (alanine transaminase). Serum biochemical analysis was conducted on a Konelab Prime biochemistry analyser (30i, Thermo Fisher Scientific Oy, Vantaa, Finland).

Chlamydial detection was conducted using an established qPCR [44] for chlamydial 23S, *ompB* and koala *beta-actin* as a sample quality control, following DNA extraction on a magnetic bead-based 96-well extraction system (Kingfisher Flex, Thermo Fisher Scientific) with positive and negative controls.

### 2.5. Genetic Sample Extraction and Sequencing

As a part of the koala whole genome survey, genomic analysis was conducted on 20 koalas from the Snowy Monaro Shire [45]. DNA was extracted from 200 µL of whole blood from koalas in Peak View (residents in the burnt: *n* = 5, rehabilitated: *n* = 6) and Numeralla (residents in the unburnt: *n* = 9) using the standard MagAttract HMW DNA kit (QIAGEN, Hilden, Germany) protocol with elution in 60 µL buffer AE. The quantity of DNA was determined by Qubit 2.0 (Thermo Fisher Scientific) and the quality was assessed through 0.8% agarose TBE gel stained with SYBR Safe (Life Technologies, Thermo Fisher Scientific), where 2 µL of DNA was stained with 4 µL of 10% loading dye (Bioline, London, UK). DNA was separated alongside a 1-kb size standard (Bioline) for 30 min at 90 V, bands were visualised using a Gel Doc XR + (Bio-Rad, Hercules, CA, USA) under ultraviolet light and images were analysed with ImageLab (Bio-Rad). DNA samples underwent additional QC, Illumina DNA PCR-free library preparation (Illumina, San Diego, CA, USA) and whole genome sequencing (WGS) at the Ramaciotti Centre for Genomics (UNSW). Samples were sequenced as 150 bp paired ends on a Novaseq S4 flowcell obtaining ~30× coverage per sample. Resequencing data from the 20 koalas were aligned with the reference genome (GCA_002099425.1_phaCin_unsw_v4.1_genomic.fasta (Johnson et al., 2018)) and variants were called using the DRAGEN Germline platform v3.8.4 (Illumina) [46]. Joint genotyping across all samples was performed using DRAGEN JointGenotyping v3.8.4.

### 2.6. Population Genetic Analysis

Variants were restricted to biallelic SNPs using GATK v4.2.0.0 [47], any sites with missing data were removed using vcftools v0.1.14 [48]. SNPs were further filtered on a minor allele frequency (MAF) of 0.08 to ensure an SNP was seen in at least 2 individuals (3/2N). Linked SNPs were removed with PLINK v1.90 [49] using an R^2^ of 0.4, a window size of 50 SNPs and a step of five SNPs. Using R v4.1.1 [50], the vcf file was then transformed to a genind object using the adegenet package v2.4.1 [51] and a PCoA analysis was conducted. Using the set of SNPs filtered only on missing data, we estimated the runs of homozygosity using the homozyg function in PLINK. We used a sliding window of 50 SNPs with regions considered as homozygous if they were at least 100 kb and 100 SNPs. We allowed for one heterozygous SNP per window and five SNPs per ROH. In order to call a run of homozygosity (ROH), we required at least one SNP per 50 kb and the maximum gap between two SNPs was 200 kb and at least 5% of windows were required to contain a homozygous SNP to be considered an ROH. Heterozygosity and Fis were calculated using the populations command in Stacks v2.2 [52] using biallelic sites only with no additional filtering as to not bias heterozygosity estimates.

### 2.7. Data Analysis

Four of the biochemical and haematological parameters (urea, AST, ALT and MCV) were log transformed prior to statistical analysis as they were not normally distributed. We used the lme4 package (version 1.1.31) [53] in R (v3.6.3) [50] to test whether body condition and each of the biochemical and haematological parameters differed between koala groups and/or health checks. For each model, the health parameter was the dependent variable and koala group, health check and the interaction between the two were the fixed effects. We also included the individual koala identifier as a random effect because some koalas received multiple health checks. If effects were significant (*p* < 0.05), we used the emmeans package (v1.6.3) [54] to conduct pairwise *t*-tests to determine which treatments differed from others. We used the same packages to test for differences in each health parameter between koalas that tested either positive or negative to Chlamydia. We did not test for differences in infection rates with ocular Chlamydia due to the small number of positive cases. Urogenital Chlamydia status was a fixed effect and individual koala was a random effect. No haematological data were available for any rehabilitated koalas for the recapture check due to unexpected degradation of the blood samples during transportation. This means that haematological comparisons are only between rescue and pre-release health checks for rehabilitated koalas.

We investigated differences between rehabilitated koalas that lived or died by conducting logistic regressions of survival against individual health parameters. It was only feasible to test one health covariate at a time because of missing values in these covariates and the small number of individuals that died (*n* = 7).

## 3. Results

### 3.1. Survival Rates

Of the 56 koalas that received a health check, 10 koalas died or were euthanized. This included 7 of the 31 koalas that were rescued post-fire, with a 78% survival rate in care for the rehabilitated koalas. Post-mortems on five of these koalas showed evidence of starvation, poor body condition and a large tapeworm burden in one individual. The other two koalas were euthanized due to multiple contributing factors that made them unreleasable, such as large cysts in the reproductive tract and crepitus in the joints.

After release, two of the twelve rehabilitated koalas that were monitored with GPS collars died during the study (i.e., 17%). One sustained a bite injury two months after release, possibly from another male, and was recaptured and treatment attempted but later died. On post-mortem, a sinus was found at the left ventral thigh extending to the greater trochanter of the femur, with necrosis of the bone present. The other koala died seven months post-release, but the cause of death is unknown. No collared koalas in either the burnt or unburnt resident groups died during the study. However, an emaciated koala of advanced age with poor dental condition (worn down teeth due to age) was captured in the unburnt landscape during the pre-release checks and was euthanised due to welfare concerns.

The tracking signal on two of the GPS collars failed after deployment, but one of the koalas was able to be located again during the study and the collar was removed. Another 13 koalas dropped their collars before the completion of the study.

### 3.2. Burn Injuries

Only three out of thirty-one rescued koalas had superficial burn injuries, although there were also changes in the skin compatible with healing soft tissue injuries on the extremities of two of the rescued koalas that died. One of the burn injuries in the surviving koalas required regular bandaging and prevented that individual from climbing for a few weeks. The others were treated for burns but did not require bandaging. All three koalas made a full recovery. Interestingly, two resident koalas captured ~4 months post-fire for the monitoring study had evidence of healed burns on their footpads (patches of pale pink skin) and scars without fur around their ear margins.

### 3.3. Body Condition Score

**Figure 2:**
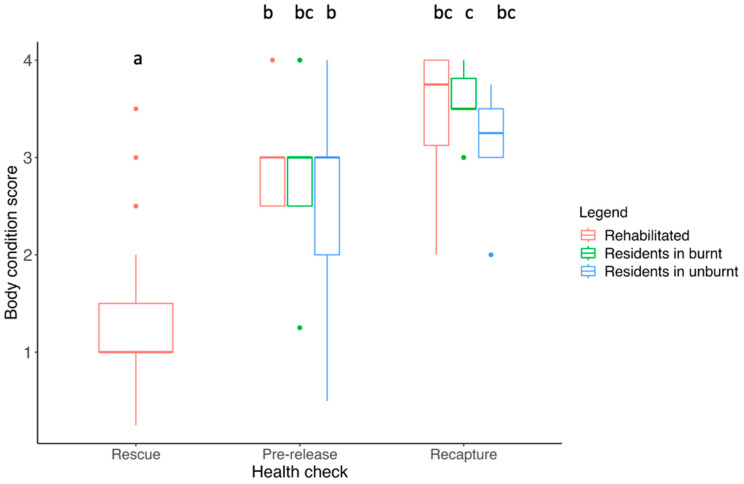
Spread of body condition scores at each health check for koalas within each group. Significance is denoted with different lettering. Note the long whisker for the residents in the unburnt at the pre-release check is due to one individual of advanced age who was in very poor condition and was subsequently euthanized on welfare grounds. The points outside the boxplots show outliers (individuals who had higher or lower BCS that fell outside the range).

At the initial health check, many of the rehabilitated koalas had a very low body condition score (BCS), with the average being 1.3 ± 0.14 (SE) out of 5. BCS differed between health checks (F_2, 79_ = 26.81, *p* < 0.001), particularly between the rescue and pre-release (*p* < 0.001) and rescue and recapture health checks (*p* < 0.001) for rehabilitated koalas (Figure 2). At the pre-release and recapture health checks, koalas from all groups were in similar body condition (*p* > 0.05; Figure 2).

### 3.4. Overall Health and Chlamydia Status

Of the 24 adult female koalas examined in this study, 13 had pouch and/or back young present between January and September 2020. This equated to 44% of female rehabilitated individuals, 40% of female burnt residents and 36% of female unburnt residents. Two rehabilitated koalas and one unburnt resident lost their pouch young during the study, while all others reared them to independence.

Only 3 of the 56 individual koalas examined tested positive for ocular *Chlamydia pecorum* at any of the three health checks (Table 2) and all three were asymptomatic juvenile individuals who were rescued from the fire grounds. Ocular *Chlamydia pecorum* was not detected in the seven koalas that presented with eye abnormalities, including discharge, corneal ulceration and opacity. In contrast, urogenital shedding of *C. pecorum* was detected in 31 of 56 koalas (55%; Table 2). This included the mothers of two of the juveniles with ocular chlamydial shedding. None of the koalas involved in the post-release monitoring program were treated for chlamydiosis even if they tested positive. Koalas in which urogenital chlamydial shedding was detected at their first health check were also positive at other health checks. Likewise, koalas that were negative tested negative throughout the study. Signs of disease typically associated with urogenital *C. pecorum* were also very low, with 48/56 koalas at their first health check having a rump score of 0/7, which indicates normal fur, normal cloaca and no evidence of wet bottom [55]. A total of seven koalas scored 1/7 (slight discoloration and mild urine leakage). The highest rump score was 3/7 for one female koala in the unburnt area, which indicates discoloration of the tail area fur and stronger ‘wet bottom’ smell. For all koalas that received a health check at recapture, eight scored 0/7, nine scored 1/7 and one scored 1.5/7.

### 3.5. Blood Biochemistry

**Figure 3:**
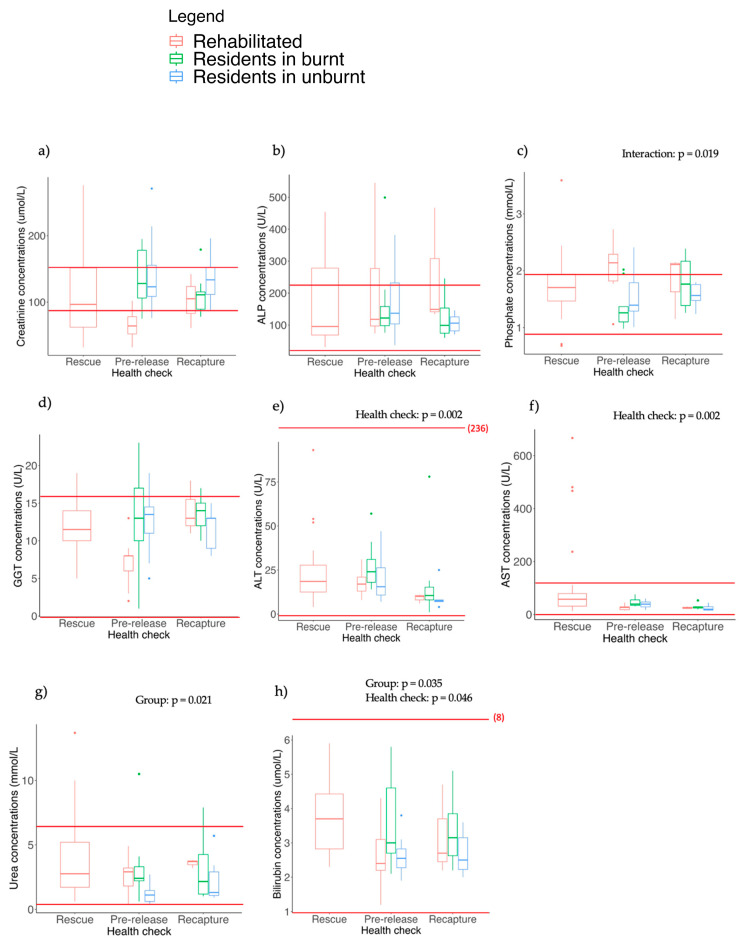
Spread of data within groups and health checks for each biochemical parameter. Each biochemical parameter is represented by a letter from (**a**–**h**). Red lines indicate the reference range. *p*-values are provided where differences between koala groups, health checks or the interaction were significant. Refer to the text for the pairwise comparisons. The points outside the boxplots show outliers (individuals who had higher or lower values for each parameter that fell outside the range).

There were no significant differences detected between serum creatinine (Figure 3a) and ALP (Figure 3b) concentrations for different koala groups or health checks (*p* > 0.05 for all tests). While we observed some differences in blood biochemistry results between koala groups and/or health checks for other parameters (described below), most individuals were within the published reference ranges [9,56] (Figure 3).

For phosphate, there was a significant interaction between group and health check (F_2, 19.73_ = 4.86, *p* = 0.019), likely because phosphate concentrations decreased between the pre-release and recapture checks in rehabilitated koalas but increased in the resident koalas in burnt habitats (Figure 3c). A post hoc analyses showed that, at the pre-release check, phosphate levels were significantly higher (and above the reference range for many individuals) in rehabilitated koalas compared to koalas in the burnt (*p* < 0.001) and unburnt (*p* = 0.003) resident groups (Figure 3c). There was also a near significant group x health interaction for GGT (F_2, 27.26_ = 3.24, *p* = 0.054; Figure 3d), with mean values lower at pre-release compared to recapture in rehabilitated koalas but not in other groups. Furthermore, GGT concentrations were lower for koalas in the rehabilitated group at the pre-release check relative to all other checks for all koala groups (*p* < 0.05 for all pairwise comparisons) but were within the reference range (Figure 3d).

There were significant differences in the concentrations of both ALT (F_2, 36.88_ = 7.15, *p* = 0.002; Figure 3e) and AST between health checks (F_2, 18.46_ = 8.65, *p* = 0.002; Figure 3f). ALT concentrations were significantly lower at the final recapture check compared to the pre-release check in both residents in the burnt (*p* = 0.008) and unburnt landscape (*p* = 0.025; Figure 3e). For AST, concentrations were significantly higher in rehabilitated koalas at the rescue check compared to pre-release (*p* = 0.003; Figure 3f).

There was a significant difference in urea concentrations between koala groups (F_2, 55.15_ = 4.13, *p* = 0.021), with resident koalas in the unburnt landscape having significantly lower concentrations of urea at the pre-release check compared to residents in the burnt landscape (*p* = 0.005) and rehabilitated koalas (*p* = 0.016; Figure 3g).

There were also differences in bilirubin concentrations between koala groups (F_2, 61.05_ = 3.54, *p* = 0.035) and health checks (F_2, 36.41_ = 3.36, *p* = 0.046; Figure 3h). Bilirubin levels were higher in residents in the burnt landscape at the pre-release check compared to both rehabilitated koalas (*p* = 0.02) and residents in the unburnt landscape (*p* = 0.007; Figure 3h). Bilirubin concentrations were also higher in rehabilitated koalas at rescue compared to pre-release (*p* = 0.002; Figure 3h).

For most biochemical parameters, values were similar regardless of whether koalas tested positive or negative to urogenital *C. pecorum*. The exceptions were for ALP (F_1, 70.38_ = 11.85, *p* < 0.001) and phosphate (F_1, 64.711_ = 6.36, *p* = 0.014), with concentrations for both constituents lower in koalas that tested positive to urogenital chlamydia.

We found a statistically significant negative association between survival and the log of ALT, suggesting that koalas with high ALT were less likely to survive. Although statistically significant, this finding is considered tentative due to the small number of observations involved (only five koalas where ALT was measured died).

### 3.6. Haematology

**Figure 4:**
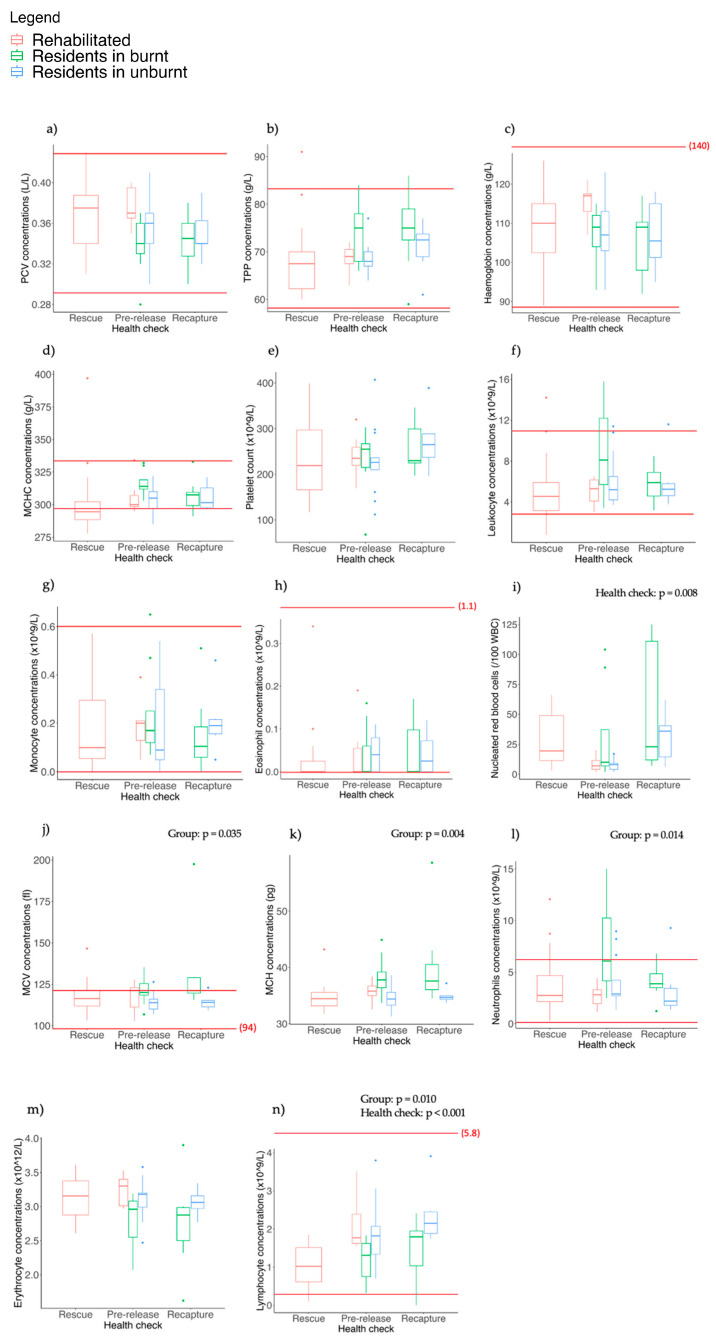
Spread of data within groups and health checks for each haematological parameter. Each haematological parameter is represented by a letter from (**a**)–(**n**). Red lines indicate reference range. *p*-values are provided where differences between koala groups, health checks or the interaction were significant. Refer to the text for the pairwise comparisons. The points outside the boxplots show outliers (individuals who had higher or lower values for each parameter that fell outside the range).

Similar to the blood biochemistry, the haematological results for most individuals were within the published reference ranges [9,56] (Figure 4). There were no significant differences between the koala groups or the health checks for PCV, TPP, haemoglobin, MCHC, platelet count, leukocytes, monocytes, eosinophils or basophils (*p* > 0.05 for all tests; Figure 4a–h). A figure for basophils has not been included as most values were 0.

Concentrations of nucleated red blood cells (NRBCs) differed between health checks (F_2, 27.35_ = 5.79, *p* = 0.008; Figure 4i) and were significantly higher at the pre-release check for residents in the burnt landscape compared to residents in the unburnt landscape (*p* = 0.039). NRBCs were also significantly higher at the recapture check compared to the pre-release check in residents in the unburnt landscape (*p* = 0.048).

There were significant differences between koala groups for MCV (F_2, 59.53_ = 3.54, *p* = 0.035; Figure 4j), MCH (F_2, 59.70_ = 6.003, *p* = 0.004; Figure 4k) and neutrophils (F_2, 48.48_ = 4.65, *p* = 0.014; Figure 4l), and there was also a trend for erythrocytes to differ between groups (F_2, 59.25_ = 3.006, *p* = 0.057; Figure 4m). MCV was significantly higher at the recapture health check compared to the pre-release check for residents in the burnt landscape (*p* = 0.045) and significantly higher at recapture in residents in the burnt landscape compared to residents in the unburnt landscape (*p* = 0.011; Figure 4j). MCH was significantly higher at both the pre-release and recapture checks in residents in the burnt landscape compared to residents in the unburnt landscape (*p* = 0.011 and *p* = 0.002, respectively; Figure 4k). At the pre-release check, neutrophils were above the reference range in almost half of the residents in the burnt landscape and were higher on average than both residents in the unburnt landscape (*p* = 0.01) and rehabilitated koalas (*p* = 0.003; Figure 4l). However, at recapture, neutrophils for residents in the burnt landscape were significantly lower than at pre-release (*p* = 0.036) and most were within the reference range (Figure 4l).

There were significant differences in lymphocytes for both koala groups (F_2, 55.43_ = 4.96, *p* = 0.01) and health checks (F_2, 24.91_ = 13.46, *p* < 0.001; Figure 4n). At pre-release, lymphocytes in residents in the burnt landscape were significantly lower than both unburnt residents (*p* = 0.011) and rehabilitated koalas (*p* = 0.008) during this check. At recapture, lymphocytes were also significantly lower in burnt residents compared to unburnt residents (*p* = 0.05). Finally, in rehabilitated koalas, lymphocyte values were significantly higher at pre-release compared to their first health check at rescue (*p* < 0.001). There were no significant differences detected in either koala group or health check for the neutrophil/lymphocyte ratio.

There were no significant differences observed between any of the haematological parameters and Chlamydia status, or between koalas that lived or died (*p* > 0.05 for all tests).

### 3.7. Genetic Analysis

All 20 samples passed quality control and were sequenced with an average 36.4X coverage (range 29.2–49.3) with a total of 18,953,300,000 paired end reads. After filtering on missingness and MAF, there were 9,988,662 SNPs, and after removing linked SNPs there were 948,924 SNPs for downstream analysis.

**Figure 5:**
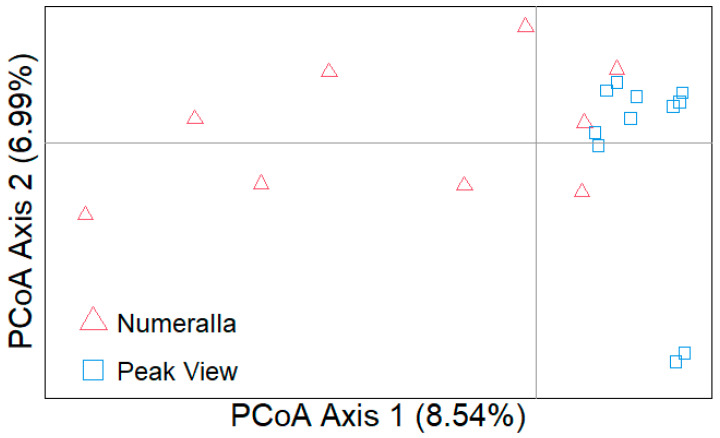
PCoA plot based on 948,924 biallelic SNPs with individuals from two koala groups in the Snowy Monaro region (Numeralla and Peak View, NSW).

**Figure 6:**
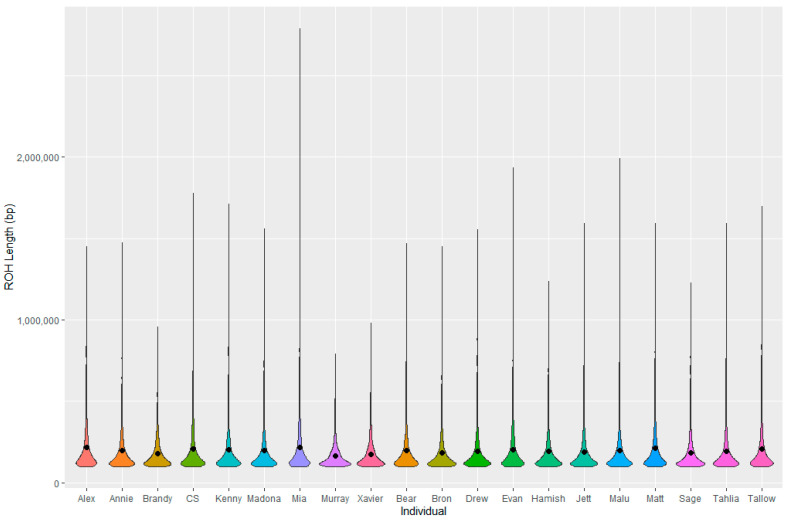
Distribution of runs of homozygosity (ROH) for each individual.

PCoA showed minimal population structuring among individuals from Peak View and Numeralla, with the majority of individuals clustering together (Figure 5). Observed heterozygosity was similar between the two groups (Numeralla 0.172 ± 0.053, Peak View 0.174 ± 0.053), as was expected heterozygosity (Numeralla: 0.157 ± 0.035, Peak View: 0.160 ± 0.035). Fis for both populations was close to zero (Numeralla: −0.005 ± 0.047, Peak View: −0.0091 ± 0.047). The majority of the ROH were small with the average length of ROH at 196 kbp across all individuals, with the longest ROH identified as 2.79 Mbp (Figure 6).

## 4. Discussion

This is the first research on the impacts of bushfires and rehabilitation and release into bushfire-affected areas on the overall health, chlamydial shedding and biochemical and haematological parameters of koalas. This previous knowledge gap hampered decisions about whether to return koalas rescued from fire-affected areas to burnt habitats or whether to translocate them to nearby unburnt habitats. Our results showed that body condition significantly improved in rehabilitated koalas over time, and they were in similar body condition to koalas living in fire-affected and unburnt landscapes at their time of release. Unexpectedly, there was no difference in body condition between koalas in burnt and unburnt habitat, suggesting there was a sufficient quantity and quality of food available to sustain their body conditions in burnt areas. However, more studies are needed to determine whether this pattern is consistent across other koala populations post-fire. While there were detectable differences in some of the measured biochemical and haematological parameters between koala groups and/or health checks, there was no evidence that fire and rehabilitation had any consistent biologically significant effects on the general health of koalas or on the signs or prevalence of disease. Thus, our study suggests that, within the study timeframe, the health of rehabilitated and resident koalas in burnt habitats was not disadvantaged compared to koalas in unburnt habitats.

### 4.1. Body Condition and Overall Health

As expected, koalas rescued from fire grounds were in very poor body condition, with an initial mean score of 1.3 out of 5, where 1–2 is considered emaciated or poor condition, 3 is healthy/adequate and 4–5 is very good/excellent [9,41,42,43,55]. After their time in care, the BCS of koalas in the rehabilitated group had increased and was similar to resident koalas in the burnt and unburnt group at both the pre-release and final recapture health checks. Koalas in care and after release therefore did not gain or lose body condition at a substantially different rate than koalas who remained and survived in the wild. This means that within four months of fire there were sufficient resources in burnt habitats to support healthy koalas. It also may be worthwhile considering other measures of health prior to release, as Leigh et al. [57] found that both BCS and climbing ability were important determinants of acute survival following release from rehabilitation, with koalas with stronger climbing abilities and higher body condition scores more likely to survive. This suggests that release should be considered if suitable habitat is present and koalas are in good physical condition.

Landscapes typically burn with a mosaic of fire severity due to variation in weather and topography, and the fire in the study region was no exception. Thus, fire severity ranged from low (only understorey burnt) to extreme (full canopy consumption) in patches across the burnt area inhabited by koalas in our study. We do not know whether the koalas in our study relied on trees with intact canopies or whether they extensively utilised the widespread epicormic regrowth. It is likely that they used both given that rehabilitated koalas were willing to eat epicormic regrowth from some eucalypt species alongside mature foliage while in care (Lane et al. submitted). Nevertheless, future studies should attempt to better understand the value of epicormic growth as food for koalas and how it may shape the way that koalas use fire-impacted landscapes and their health.

Drought conditions are known to negatively impact koala populations, affecting mortality rates, breeding success and dispersal of individuals [58,59]. It is possible that the initial poor condition and some of the wide variation in values recorded in the biochemical and haematological parameters in the rescued koalas could be due to the extreme drought conditions and heat that were prominent in the lead up to the fire in addition to fire effects. For example, several rehabilitation groups reported that drought conditions had greater impacts on the koalas that came into care during 2019–2020 than the fires [8]. Droughts and heatwaves can trigger leaf fall and foliage damage, causing a reduction in the availability of sufficient food, ultimately leading to malnutrition, dehydration and possible death in koalas [60,61]. A reduction in available browse also creates other indirect consequences for koalas, including increased competition, lower breeding success and higher susceptibility to disease [62]. These drought events can also compound the effects of fire and other habitat-altering events (e.g., logging) [63], making koalas weaker and more susceptible when there is a major bushfire event. It is important to consider that with a changing climate [26], more koalas may be displaced or disrupted by both droughts and fire. Understanding how their physiology is affected will be important for future rehabilitation efforts and knowing when to release them.

### 4.2. Burn Injuries

The number of fire-related injuries observed in koalas rescued from the Monaro region was relatively low (only three of thirty-one) compared to rescue efforts in other areas. For example, Parrott et al. [7] reported that 20% of koalas rescued in Victoria during the megafires were euthanised due to fire-related injuries. Likewise, 67.4% of koalas that came into triage units on Kangaroo Island, South Australia, were suffering from burn injuries, and 45.6% died or needed to be euthanised [9]. The lower numbers in our study may have been due to a combination of relatively low encounter rates between searchers and koalas due to low densities [33], higher mortality rates from intense fires or difficulties with access to remote areas and restricted fireground access. Despite this, the results from all three studies demonstrate that many more koalas would likely have perished without the necessary care provided by veterinary teams and wildlife carers post-fire. Our findings provide additional data to show that koalas who survive the initial fire event and are later released can live and maintain health in a burnt landscape.

### 4.3. Chlamydia Status

In this study, shedding of *C. pecorum,* an intracellular bacterium that is widespread throughout most koala populations [23,64], was detected in 59% of koalas. Interestingly, very few of the Monaro koalas exhibited clinical signs of disease during any health check, with only one koala in the rehabilitation group euthanised and two requiring antibiotic treatment due to the presence of mild paraovarian cysts. Furthermore, 44% of the adult females who were infected with *C. pecorum* in our study had pouch or back young. These findings place the Monaro koalas in line with other populations that appear to have a low prevalence of clinical disease. *C. pecorum* can cause a range of diseases, with the most common being conjunctivitis and urogenital disease, the latter of which can lead to infertility in females [15,24,58,65,66]. Koalas from the Monaro region showed higher rates of chlamydial shedding and urogenital disease relative to ocular sites, which is consistent with other populations in both Queensland [67,68] and Victoria [42]. *C. pecorum* has also been detected in most koala populations, with the prevalence and severity of *C. pecorum* varying significantly between populations [23,64], infecting individuals without causing outward and obvious symptoms or disease [69]. For example, in a koala population in south-eastern Queensland, 71% of koalas tested positive for *C. pecroum*; however, only 9% of individuals showed overt clinical disease [70]. Furthermore, 82% of females had either back young or pouch young, despite 67% being infected [70]. In another Queensland population, of the 67% of males that tested positive to urogenital *Chlamydia*, only one-third showed clinical symptoms [64]. A low prevalence of clinical disease has also been observed in South Australian populations including in the Mount Lofty Ranges and at Cleland Wildlife Sanctuary [23].

Rates of chlamydial shedding were similar between rehabilitated koalas and resident koalas in burnt and unburnt habitats. Furthermore, we did not observe any changes either in the presence of chlamydial infection or the clinical signs of disease in individuals across the course of the study, even though environmental stressors such as bushfire have the potential to exacerbate clinical disease [25]. These results further support the idea that neither fire nor rehabilitation have major effects on chlamydial status in koalas in the Snowy Monaro region. It also supports the idea that there were limited negative health impacts of releasing rehabilitated koalas into burnt habitats in the Monaro region, even with untreated chlamydial infections. It is important to note, however, that *C. pecorum* is primarily sexually transmitted in koalas [23,24], although it can also be transmitted from mother to young during pap feeding [23,67]. Sexual transmission of Chlamydia may potentially be lower in burnt landscapes, as there may be fewer individuals to interact with due to lower densities. This has not been tested but should be considered.

### 4.4. Genetics

Using a whole genome sequencing (WGS) approach, we assessed the genetic health of koalas from the Monaro region of NSW and report the first genetic analysis of this population of koalas. We show that this population of koalas displays diversity comparable to other populations of koalas [71,72]. An analysis of ROH is able to give insights into inbreeding history of an individual, with long ROH segments reflecting recent inbreeding events and shorter ROH segments reflecting historical inbreeding that has been broken by recombination [73]. The majority of the ROH were <0.4 MBp in length and only eight ROH segments were larger than 1.6 MBp, indicating some recent inbreeding in those individuals. On average, the lengths of ROH in this koala population were lower than other wildlife species including killer whales and rhinoceros [74,75]. Population structuring indicates higher variation amongst individuals from Numeralla with some mixing between the two sites, which is to be expected as the sites are only 15 km apart. The WGS data here provide a baseline with which to compare the genetic health of Monaro koalas in the future and also add to the growing body of genomic data becoming available for koalas.

### 4.5. Biochemical and Haematological Parameters

Although we detected some differences in haematological and biochemical parameters between koala groups, health checks, Chlamydia status and age classes, values were predominantly within the published reference ranges [9,56]. While this suggests that the differences noted are unlikely to be biologically meaningful, perhaps reflecting normal variation between and within individuals, it should also be considered that wildlife reference ranges are generated on small sample sizes and either for captive koalas of known health status or wild koalas, the health and stress status of which can only be partially assessed, and so may be broader than true ranges.

Most of the differences observed between biochemical and haematological parameters cannot be easily explained, but some results provide possible insight into the effects of fire and rehabilitation on koala health. The first is for urea. Blood urea and creatinine levels assist with assessment of renal function [76,77,78]. Both rehabilitated koalas and residents in the burnt landscape had higher concentrations of urea 4–6 months after fire at the pre-release check compared to residents in the unburnt area, but the difference disappeared by the final recapture check 8–9 months later. There could be several reasons for this. Firstly, koalas living in the burnt area may be more dehydrated due to ingestion of leaves with a lower moisture content compared to those in the unburnt area. However, preliminary data suggest that post-fire epicormic regrowth on eucalypts contains more moisture than mature leaves in the canopy [79]. Furthermore, since creatinine, which can be used with urea to assess dehydration, was not elevated in burnt residents at the pre-release health check and values were mostly within the reference range for both urea and creatinine, the results may not indicate dehydration. An alternative may be related to urea as an indicator of protein status and thus nutrition [80]. For example, low creatinine and urea concentrations observed in tammar wallabies (*Macropus eugenii*) were linked to nutritional stress [80]. Similarly, red-necked pademelons (*Thylogale thetis*) had higher plasma urea levels with a higher consumption of nitrogen in the diet [81]. The epicormic regrowth from some eucalypt species can contain relatively high concentrations of nitrogen (a proxy for protein) (Lane et al. submitted). It is plausible that koalas in care or living in burnt habitat may have been eating diets higher in protein prior to their pre-release check and that this may have contributed to the difference in urea status. Future studies could investigate links between dietary protein intake and levels of urea in koalas.

A number of enzymes found predominantly in the liver can be indicators of hepatocellular damage and disease [82,83,84,85,86]. These include AST, ALT, ALP and GGT. These enzymes can be non-specific and so should be observed in conjunction with one another and with clinical signs [87,88]. Elevated levels of AST and GGT have been associated with lymphosarcoma and liver disease in koalas [56,89], as well as diabetes mellitus [90] and oxalate nephrosis, a prevalent disease in South Australian koalas [91]. A study on chlamydiosis in koalas also found elevated AST and ALT levels; however, this was during treatment with subcutaneous injections and the results could potentially have been explained by localised myopathy [92]. We found no differences in AST and ALT levels between Monaro koalas that tested either negative or positive for *C. pecorum.* Furthermore, mean values for all liver enzymes were within the reference ranges for both negative and positive cases of Chlamydia. This may not be surprising given that most infections were subclinical.

Elevated AST can also be attributed to haemolysis and liver and muscle damage [93,94]. It is likely that the elevated levels in rehabilitated koalas at their rescue health check were associated with impacts of poor nutrition and stress metabolism on hepatocyte integrity, rather than primary liver disease. Elevated AST has been associated with stress in a variety of marsupials, including capture stress in western ringtail possums (*Pseudocheirus occidentalis*) [19], trauma from fighting in hairy-nosed wombats (*Lasiorhinus latifrons*) [95] and oxalate nephrosis [91]. Bilirubin, a bile pigment formed when haemoglobin breaks down and important in liver metabolism [96,97,98], was higher in rehabilitated koalas when they came into care compared to when they were released. In horses, which are hindgut fermenters like koalas, elevated bilirubin can be associated with fasting [99]. It is possible that this could also have been an indication that rescued koalas did not have access to sufficient food in the wild.

Leukocytes (white blood cell count (WBC)) form a major part of the immune system [100], and include lymphocytes, neutrophils, monocytes, eosinophils and basophils [100]. While differences were detected in lymphocyte and neutrophil concentrations, most values were within reference ranges, and the higher values observed in some resident koalas in the burnt area at pre-release were within the reference range at recapture. The neutrophil/lymphocyte ratio (N:L) is sometimes used to assess inflammatory response [101,102], with an elevated neutrophil/lymphocyte ratio associated with stress and illness in laboratory animals and domestic pets [103,104,105]. In this study, we found no significant differences between koala groups or health checks for the N:L ratio. Although linked to stress and disease, Canfield and O’Neill [56] found that neutrophil and lymphocyte proportions and ratios can be highly variable in healthy koalas.

Variation was also observed in nucleated red blood cells (NRBCs), with significant differences detected between health checks, though the reason for this is unclear. NRBCs are immature red blood cells [106] and their presence in the bloodstream can be associated with bone marrow damage and a number of diseases in people [106,107]. In koalas, however, NRBCs are relatively common and are not always indicative of disease [56,108,109,110]. Reference ranges for NRBCs have been reported to be between 0 and 8–10 for koalas [110,111]. Higher NRBCs have been linked with disease in koalas, for example, Fabijan et al. [112] found that koalas with neoplasia had significantly higher counts of NRBCs and koalas with ocular Chlamydia also had higher NRBC counts compared to those who had urinary tract and reproductive disease, though the mechanism for this is unclear. Higher counts of NRBCs have also been documented in koalas with anaemia [110]. The values observed in the koalas from the Southern Tablelands are much higher and more variable than reports from other studies. While this could suggest underlying health issues, their body condition and other results suggest that they are otherwise in a good health condition. While the presence of NRBCs in these koalas could suggest regenerative anaemia, young koalas can also have higher counts of NRBCs.

## 5. Conclusions

This study provides important baseline health data for the Snowy Monaro population of koalas. Koalas in the Monaro region of NSW appear to be in good health, with low prevalence of clinical Chlamydia and relatively high proportions of adult females with joeys. The study population had adequate to excellent body condition by the completion of the project, even though 19 of the 32 tracked koalas were living in burnt habitats. From a management perspective and given the health status of koalas in this region, it is important to try and conserve this seemingly healthy population and the valuable habitat that can support them. Due to the steep and rocky terrain, it is unlikely to be an area that will be developed in the future, and, therefore, conserving this population may be more promising.

This is the first study to demonstrate that the general health indicators of koalas living in burnt habitat between 4 and 16 months after fire were similar to koalas living in unburnt habitat during the same time period. The health and trajectory of koalas released into burnt habitats after rehabilitation mirrored that of koalas that remained in situ after fire in both burnt and unburnt habitats. These findings suggest that, in the Monaro, uninjured koalas do not need to be removed from burnt habitats and rehabilitated koalas can be released into fire-impacted areas, provided there is access to patches of trees with canopy cover or epicormic growth. However, more research is needed in this area to determine at what point there is too little food, and the other implications of rehabilitation, such as movements and habitat use. Many of the koalas in this study were living within the fire scar and utilising burnt areas. In the future, it would also be important to look at the home ranges of koalas and the nutritional composition of the landscape to better understand how koalas are using fire-impacted areas. Future research could also explore whether the findings from this study are consistent across other regions and koala populations. Retaining uninjured koalas in the landscape alleviates resources needed to maintain animals in care and enables them to recover in their natural environment, which may be less stressful overall. Inevitably, there will be more bushfires in the Australian landscape, and the implications of this study have direct applications to management decisions and rehabilitation efforts for koala populations post-fire.

## Figures and Tables

**Table 1:** Number of koalas given each health check from each group. The groups are rehabilitated koalas (note that only a subset of individuals was tracked post-release and therefore received a pre-release and recapture check), resident koalas (individuals caught, collared and released on the same day) in burnt habitat and resident koalas in unburnt habitat (see Table S1 for a full list of the individual koalas used in this study).

Health Check (Timeframe)	Rehabilitated	Non-Rescued Residents in the Burnt Habitat	Non-Rescued Residents in the Unburnt Habitat
**Rescue** (23 January 2020–13 March 2020)	31	NA	NA
**Pre-release** (25 May 2020–18 December 2020)	12	9	16
**Recapture** (5 February 2021–16 May 2021)	4	8	6

**Table 2:** Number of koalas shedding *Chlamydia pecorum* in each group.

	Number of Individuals with Ocular Chlamydia Only	Number of Individuals with Urogenital Chlamydia Only	Number of Individuals with both Ocular and Urogenital Chlamydia
Rehabilitated koalas (*n* = 31)	2	17	1
Residents in the burnt habitat (*n* = 9)	0	4	0
Residents in the unburnt habitat (*n* = 16)	0	10	0

## Data Availability

All raw fastq sequences, aligned bam files and metadata for koala genomes from the Koala Genome Survey are available at https://awgg-lab.github.io/australasiangenomes/species/Phascolarctos_cinereus.html (accessed on 4 July 2020). Raw data used for analyses of health parameters, body condition and Chlamydia status is online at the Australian National University Data Commons Library.

## Supplementary Materials

The following supporting information can be downloaded at: https://www.mdpi.com/article/10.3390/ani13182863/s1. Table S1: list of all koalas including group, health check, sex, tooth wear class, presence of pouch young and outcome.

## Appendix A

A1: sampling of koalas following capture and restraint from the Koala Health Hub: https://drive.google.com/file/d/1MyOOQwXsIfAZAVwipCDv2NwYuMXTy9XU/view (accessed on 25 May 2020).

## References

[1] Attiwill P.M., Adams M.A. (2013). Mega-fires, inquiries and politics in the eucalypt forests of Victoria, south-eastern Australia. For. Ecol. Manag..

[2] Penman T.D., Kavanagh R.P., Binns D.L., Melick D.R. (2007). Patchiness of prescribed burns in dry sclerophyll eucalypt forests in south-eastern Australia. For. Ecol. Manag..

[3] Russell-Smith J., Yates C.P., Whitehead P.J., Smith R., Craig R., Allan G.E., Thackway R., Frakes I., Cridland S., Meyer M.C.P. (2007). Bushfires ‘down under’: Patterns and implications of contemporary Australian landscape burning. Int. J. Wildland Fire.

[4] Wintle B.A., Legge S., Woinarski J.C. (2020). After the megafires: What next for Australian wildlife?. Trends Ecol. Evol..

[5] NSW Department of Planning Industry and Environment (2020). NSW Fire and the Environment 2019-20 Summary.

[6] Van Eeden L.M., Nimmo D., Mahony M., Herman K., Ehmke G., Driessen J., O’Connor J., Bino G., Taylor M., Dickman C. (2020). Impacts of the Unprecedented 2019–2020 Bushfires on Australian Animals.

[7] Parrott M.L., Wicker L.V., Lamont A., Banks C., Lang M., Lynch M., McMeekin B., Miller K.A., Ryan F., Selwood K.E. (2021). Emergency response to Australia’s black summer 2019–2020: The role of a zoo-based conservation organisation in wildlife triage, rescue, and resilience for the future. Animals.

[8] Lunney D., Cope H., Sonawane I., Haering R. (2022). A state-wide picture of koala rescue and rehabilitation in New South Wales during the 2019–2020 bushfires. Aust. Zool..

[9] Dunstan E., Funnell O., McLelland J., Stoeckeler F., Nishimoto E., Mitchell D., Mitchell S., McLelland D.J., Kalvas J., Johnson L. (2021). An analysis of demographic and triage assessment findings in bushfire-affected koalas (*Phascolarctos cinereus*) on Kangaroo Island, South Australia, 2019–2020. Animals.

[10] Menkhorst P., Ramsey D., O’Brien T., Hynes E., Whisson D. (2019). Survival and movements of koalas translocated from an over-abundant population. Wildl. Res..

[11] Whisson D.A., Holland G.J., Carlyon K. (2012). Translocation of overabundant species: Implications for translocated individuals. J. Wildl. Manag..

[12] Lunney D., Gresser S., O’neill L.E., Matthews A., Rhodes J. (2007). The impact of fire and dogs on koalas at Port Stephens, New South Wales, using population viability analysis. Pac. Conserv. Biol..

[13] Zylstra P. (2019). Fire Regimes for Risk Management of Koalas on the NSW Southern Tablelands.

[14] Crowther M.S., Lunney D., Lemon J., Stalenberg E., Wheeler R., Madani G., Ross K.A., Ellis M. (2014). Climate-mediated habitat selection in an arboreal folivore. Ecography.

[15] Martin R., Handasyde K.A. (1999). The Koala: Natural History, Conservation and Management.

[16] Matthews A., Lunney D., Gresser S., Maitz W. (2016). Movement patterns of koalas in remnant forest after fire. Aust. Mammal..

[17] Burrows G. (2002). Epicormic strand structure in Angophora, Eucalyptus and Lophostemon (Myrtaceae): Implications for fire resistance and recovery. New Phytol..

[18] Burrows N. (2008). Linking fire ecology and fire management in south-west Australian forest landscapes. For. Ecol. Manag..

[19] Clarke J., Warren K., Calver M., de Tores P., Mills J., Robertson I. (2013). Hematologic and serum biochemical reference ranges and an assessment of exposure to infectious diseases prior to translocation of the threatened western ringtail possum (*Pseudocheirus occidentalis*). J. Wildl. Dis..

[20] Waters D.A., Burrows G.E., Harper J.D. (2010). Eucalyptus regnans (Myrtaceae): A fire-sensitive eucalypt with a resprouter epicormic structure. Am. J. Bot..

[21] Williams P.R., Parsons M., Jensen R., Tran C. (2012). Mechanisms of rainforest persistence and recruitment in frequently burnt wet tropical eucalypt forests. Austral Ecol..

[22] McCallum H., Kerlin D.H., Ellis W., Carrick F. (2018). Assessing the significance of endemic disease in conservation—Koalas, chlamydia, and koala retrovirus as a case study. Conserv. Lett..

[23] Polkinghorne A., Hanger J., Timms P. (2013). Recent advances in understanding the biology, epidemiology and control of chlamydial infections in koalas. Vet. Microbiol..

[24] Quigley B.L., Timms P. (2020). Helping koalas battle disease–Recent advances in Chlamydia and koala retrovirus (KoRV) disease understanding and treatment in koalas. FEMS Microbiol. Rev..

[25] Albery G.F., Turilli I., Joseph M.B., Foley J., Frere C.H., Bansal S. (2021). From flames to inflammation: How wildfires affect patterns of wildlife disease. Fire Ecol..

[26] Lucas C., Hennessy K., Mills G., Bathols J. (2007). Bushfire Weather in Southeast Australia: Recent Trends and Projected Climate Change Impacts.

[27] Grogan L.F., Peel A.J., Kerlin D., Ellis W., Jones D., Hero J.-M., McCallum H. (2018). Is disease a major causal factor in declines? An evidence framework and case study on koala chlamydiosis. Biol. Conserv..

[28] Gonzalez-Astudillo V., Allavena R., McKinnon A., Larkin R., Henning J. (2017). Decline causes of Koalas in South East Queensland, Australia: A 17-year retrospective study of mortality and morbidity. Sci. Rep..

[29] McAlpine C., Lunney D., Melzer A., Menkhorst P., Phillips S., Phalen D., Ellis W., Foley W., Baxter G., De Villiers D. (2015). Conserving koalas: A review of the contrasting regional trends, outlooks and policy challenges. Biol. Conserv..

[30] Department of Agriculture, Water and the Environment (2022). Conservation Advice for Phascolarctos cinereus (Koala) Combined Populations of Queensland, New South Wales and the Australian Capital Territory.

[31] Snowy Monaro Regional Council (2020). Snowy Monaro Draft Rural Land Use Strategy.

[32] State Government of NSW and Department of Planning and Environment (2014). Native Vegetation of the Cooma-Monaro Shire.

[33] Allen C. (2015). OEH Koala Habitat Study: Towards a Comprehensive Koala Plan of Management for North East Monaro.

[34] Leavesley A., Rhind S. (2008). Vertebrate Fauna of Monaro Plains Conservation Reserves.

[35] Office of Environment and Heritage NSW (2012). Plan of Management: Northern Monaro Reserves.

[36] Martin S., Youngentob K.N., Clark R.G., Foley W.J., Marsh K.J. (2020). The distribution and abundance of an unusual resource for koalas (*Phascolarctos cinereus*) in a sodium-poor environment. PLoS ONE.

[37] Witt R.R., Beranek C.T., Howell L.G., Ryan S.A., Clulow J., Jordan N.R., Denholm B., Roff A. (2020). Real-time drone derived thermal imagery outperforms traditional survey methods for an arboreal forest mammal. PLoS ONE.

[38] Madani G., Ashman K., Mella V., Whisson D. (2020). A review of the ‘noose and flag’method to capture free-ranging koalas. Aust. Mammal..

[39] State Government of NSW and Department of Planning and Environment (2020). Fire Extent and Severity Mapping.

[40] Martin R. (1981). Age-specific fertility in three populations of the koala, *Phascolarctos cinereus* Goldfuss, in Victoria. Wildl. Res..

[41] Legione A.R., Patterson J.L.S., Whiteley P., Firestone S.M., Curnick M., Bodley K., Lynch M., Gilkerson J.R., Sansom F.M., Devlin J.M. (2017). Koala retrovirus genotyping analyses reveal a low prevalence of KoRV-A in Victorian koalas and an association with clinical disease. J. Med. Microbiol..

[42] Patterson J.L., Lynch M., Anderson G.A., Noormohammadi A.H., Legione A., Gilkerson J.R., Devlin J.M. (2015). The prevalence and clinical significance of Chlamydia infection in island and mainland populations of Victorian koalas (*Phascolarctos cinereus*). J. Wildl. Dis..

[43] Sarker N., Fabijan J., Owen H., Seddon J., Simmons G., Speight N., Kaler J., Woolford L., Emes R.D., Hemmatzadeh F. (2020). Koala retrovirus viral load and disease burden in distinct northern and southern koala populations. Sci. Rep..

[44] Hulse L.S., Hickey D., Mitchell J.M., Beagley K.W., Ellis W., Johnston S.D. (2018). Development and application of two multiplex real-time PCR assays for detection and speciation of bacterial pathogens in the koala. J. Vet. Diagn. Investig..

[45] Hogg C.J., Silver L., McLennan E.A., Belov K. (2023). Koala Genome Survey: An Open Data Resource to Improve Conservation Planning. Genes.

[46] Miller N.A., Farrow E.G., Gibson M., Willig L.K., Twist G., Yoo B., Marrs T., Corder S., Krivohlavek L., Walter A. (2015). A 26-hour system of highly sensitive whole genome sequencing for emergency management of genetic diseases. Genome Med..

[47] McKenna A., Hanna M., Banks E., Sivachenko A., Cibulskis K., Kernytsky A., Garimella K., Altshuler D., Gabriel S., Daly M. (2010). The Genome Analysis Toolkit: A MapReduce framework for analyzing next-generation DNA sequencing data. Genome Res..

[48] Danecek P., Auton A., Abecasis G., Albers C.A., Banks E., DePristo M.A., Handsaker R.E., Lunter G., Marth G.T., Sherry S.T. (2011). The variant call format and VCFtools. Bioinformatics.

[49] Purcell S., Neale B., Todd-Brown K., Thomas L., Ferreira M.A., Bender D., Maller J., Sklar P., De Bakker P.I., Daly M.J. (2007). PLINK: A tool set for whole-genome association and population-based linkage analyses. Am. J. Hum. Genet..

[50] R Core Team (2021). R: A Language and Environment for Statistical Computing.

[51] Jombart T. (2008). adegenet: A R package for the multivariate analysis of genetic markers. Bioinformatics.

[52] Catchen J., Hohenlohe P.A., Bassham S., Amores A., Cresko W.A. (2013). Stacks: An analysis tool set for population genomics. Mol. Ecol..

[53] Bates D., Mächler M., Bolker B., Walker S. (2014). Fitting linear mixed-effects models using lme4. arXiv.

[54] Lenth R. (2021). Emmeans: Estimated Marginal Means, Aka Least-Square Means.

[55] Koala Health Hub The University of Sydney Koala Examination Form. https://drive.google.com/file/d/1rtyYUkgTcDxFZS7UH8q1-VLzHyRZE4k6/view.

[56] Canfield P., O’Neill M., Smith E. (1989). Haematological and biochemical reference values for the koala (*Phascolarctos cinereus*). Aust. Vet. J..

[57] Leigh K.A., Hofweber L.N., Sloggett B.K., Inman V.L., Pettit L.J., Sriram A., Haering R. (2023). Outcomes for an arboreal folivore after rehabilitation and implications for management. Sci. Rep..

[58] Melzer A., Carrick F., Menkhorst P., Lunney D., John B.S. (2000). Overview, critical assessment, and conservation implications of koala distribution and abundance. Conserv. Biol..

[59] Seabrook L., McAlpine C., Baxter G., Rhodes J., Bradley A., Lunney D. (2011). Drought-driven change in wildlife distribution and numbers: A case study of koalas in south west Queensland. Wildl. Res..

[60] Degabriele R. (1981). A relative shortage of nitrogenous food in the ecology of the koala (*Phascolarctos cinereus*). Aust. J. Ecol..

[61] Gordon G., Brown A., Pulsford T. (1988). A koala (*Phascolarctos cinereus* Goldfuss) population crash during drought and heatwave conditions in south-western Queensland. Aust. J. Ecol..

[62] Cristescu R.H., Gardiner R., Terraube J., McDonald K., Powell D., Levengood A.L., Frère C.H. (2023). Difficulties of assessing the impacts of the 2019–2020 bushfires on koalas. Austral Ecol..

[63] Lunney D., Stalenberg E., Santika T., Rhodes J.R. (2014). Extinction in Eden: Identifying the role of climate change in the decline of the koala in south-eastern NSW. Wildl. Res..

[64] Hulse L.S., Beagley K., Ellis W., Fitzgibbon S., Gillett A., Barth B., Robbins A., Pyne M., Larkin R., Johnston S.D. (2020). Epidemiology of Chlamydia-induced reproductive disease in male koalas (*Phascolarctos cinereus*) from southeast Queensland, Australia as assessed from penile urethral swabs and semen. J. Wildl. Dis..

[65] Cockram F., Jackson A. (1981). Keratoconjunctivitis of the koala, *Phascolarctos cinereus*, caused by Chlamydia psittaci. J. Wildl. Dis..

[66] Wan C., Loader J., Hanger J., Beagley K., Timms P., Polkinghorne A. (2011). Using quantitative polymerase chain reaction to correlate Chlamydia pecorum infectious load with ocular, urinary and reproductive tract disease in the koala (*Phascolarctos cinereus*). Aust. Vet. J..

[67] Nyari S., Waugh C.A., Dong J., Quigley B.L., Hanger J., Loader J., Polkinghorne A., Timms P. (2017). Epidemiology of chlamydial infection and disease in a free-ranging koala (*Phascolarctos cinereus*) population. PLoS ONE.

[68] White N., Timms P. (1994). Chlamydia psittaci in a Koala (*Phascolarctos cinereus*) population in south-east Queensland. Wildl. Res..

[69] Santamaria F., Schlagloth R. (2016). The effect of Chlamydia on translocated Chlamydia-naïve koalas: A case study. Aust. Zool..

[70] Weigler B.J., Girjes A.A., White N.A., Kunst N.D., Carrick F.N., Lavin M.F. (1988). Aspects of the epidemiology of Chlamydia psittaci infection in a population of koalas (*Phascolarctos cinereus*) in southeastern Queensland, Australia. J. Wildl. Dis..

[71] Kjeldsen S.R., Raadsma H.W., Leigh K.A., Tobey J.R., Phalen D., Krockenberger A., Ellis W.A., Hynes E., Higgins D.P., Zenger K.R. (2019). Genomic comparisons reveal biogeographic and anthropogenic impacts in the koala (*Phascolarctos cinereus*): A dietary-specialist species distributed across heterogeneous environments. Heredity.

[72] Kjeldsen S.R., Zenger K.R., Leigh K., Ellis W., Tobey J., Phalen D., Melzer A., FitzGibbon S., Raadsma H.W. (2016). Genome-wide SNP loci reveal novel insights into koala (*Phascolarctos cinereus*) population variability across its range. Conserv. Genet..

[73] Curik I., Ferenčaković M., Sölkner J. (2014). Inbreeding and runs of homozygosity: A possible solution to an old problem. Livest. Sci..

[74] Foote A.D., Hooper R., Alexander A., Baird R.W., Baker C.S., Ballance L., Barlow J., Brownlow A., Collins T., Constantine R. (2021). Runs of homozygosity in killer whale genomes provide a global record of demographic histories. Mol. Ecol..

[75] Liu S., Westbury M.V., Dussex N., Mitchell K.J., Sinding M.-H.S., Heintzman P.D., Duchêne D.A., Kapp J.D., Von Seth J., Heiniger H. (2021). Ancient and modern genomes unravel the evolutionary history of the rhinoceros family. Cell.

[76] Braun J.-P., Lefebvre H., Watson A. (2003). Creatinine in the dog: A review. Vet. Clin. Pathol..

[77] Finco D.R. (1997). Kidney function. Clinical Biochemistry of Domestic Animals.

[78] Yakubu M.T., Musa I.F. (2012). Liver and kidney functional indices of pregnant rats following the administration of the crude alkaloids from Senna alata (Linn. Roxb) leaves. Iran. J. Toxicol..

[79] Natural Resources Commission, NSW Government (2022). Summary Paper: Koala and Habitat Response after the 2019-20 Wildfires in North East NSW.

[80] Robert K.A., Schwanz L.E. (2013). Monitoring the health status of free-ranging tammar wallabies using hematology, serum biochemistry, and parasite loads. J. Wildl. Manag..

[81] Dellow D., Hume I. (1982). Studies on the Nutrition of Macropodine Marsupials. 2. Urea and Water Metabolism in Thylogale Thetis and Macropus Eugenii; Two Wallabies from Divergent Habitats. Aust. J. Zool..

[82] Kaplan M.M. (1972). Alkaline phosphatase. Gastroenterology.

[83] Sherman K.E. (1991). Alanine aminotransferase in clinical practice: A review. Arch. Intern. Med..

[84] Stojević Z., Piršljin J., Milinković-Tur S., Zdelar-Tuk M., Beer Ljubić B. (2005). Activities of AST, ALT and GGT in clinically healthy dairy cows during lactation and in the dry period. Vet. Arh..

[85] Whitehead M., Hawkes N., Hainsworth I., Kingham J. (1999). A prospective study of the causes of notably raised aspartate aminotransferase of liver origin. Gut.

[86] Whitfield J. (2001). Gamma glutamyl transferase. Crit. Rev. Clin. Lab. Sci..

[87] Bennett M.D., Woolford L., O’Hara A.J., Nicholls P.K., Warren K.S. (2008). Clinical chemistry values and tissue enzyme activities in western barred bandicoots (*Perameles bougainville*). Vet. Clin. Pathol..

[88] Center S.A. (2007). Interpretation of liver enzymes. Vet. Clin. N. Am. Small Anim. Pract..

[89] Spencer A.J., Canfield P.J. (1996). Lymphoid neoplasia in the koala (*Phascolarctos cinereus*): A review and classification of 31 cases. J. Zoo Wildl. Med..

[90] Hemsley S., Govendir M., Canfield P., Connolly J. (1998). Diabetes mellitus in a koala (*Phascolarctos cinereus*). Aust. Vet. J..

[91] Speight K.N., Haynes J.I., Boardman W., Breed W.G., Taggart D.A., Rich B., Woolford L. (2014). Plasma biochemistry and urinalysis variables of koalas (*Phascolarctos cinereus*) with and without oxalate nephrosis. Vet. Clin. Pathol..

[92] Griffith J.E. (2010). Studies into the Diagnosis, Treatment and Management of Chlamydiosis in Koalas. Ph.D. Thesis.

[93] Kaneko J.J., Harvey J.W., Bruss M.L. (2008). Clinical Biochemistry of Domestic Animals.

[94] Letendre C., Sawyer E., Young L.J., Old J.M. (2018). Immunosenescence in a captive semelparous marsupial, the red-tailed phascogale (*Phascogale calura*). BMC Zool..

[95] Gaughwin M., Judson G. (1980). Haematology and clinical chemistry of hairy-nosed wombats (*Lasiorhinus latifrons*). J. Wildl. Dis..

[96] Bosma P.J. (2003). Inherited disorders of bilirubin metabolism. J. Hepatol..

[97] Stocker R., Yamamoto Y., McDonagh A.F., Glazer A.N., Ames B.N. (1987). Bilirubin is an antioxidant of possible physiological importance. Science.

[98] Wolf P.L. (1999). Biochemical diagnosis of liver disease. Indian J. Clin. Biochem..

[99] Gronwall R., Mia A.S. (1972). Fasting hyperbilirubinemia in horses. Am. J. Dig. Dis..

[100] Janeway C.A., Travers P., Walport M., Shlomchik M.J. (2001). The components of the immune system. Immunobiology: The Immune System in Health and Disease.

[101] Faria S.S., Fernandes P.C., Silva M.J.B., Lima V.C., Fontes W., Freitas-Junior R., Eterovic A.K., Forget P. (2016). The neutrophil-to-lymphocyte ratio: A narrative review. Ecancermedicalscience.

[102] Forget P., Khalifa C., Defour J.-P., Latinne D., Van Pel M.-C., De Kock M. (2017). What is the normal value of the neutrophil-to-lymphocyte ratio?. BMC Res. Notes.

[103] Hickman D.L. (2017). Evaluation of the neutrophil: Lymphocyte ratio as an indicator of chronic distress in the laboratory mouse. Lab Anim..

[104] Swan M.P., Hickman D.L. (2014). Evaluation of the neutrophil-lymphocyte ratio as a measure of distress in rats. Lab Anim..

[105] Neumann S. (2021). Neutrophil-to-lymphocyte and platelet-to-lymphocyte ratios in dogs and cats with acute pancreatitis. Vet. Clin. Pathol..

[106] Constantino B.T., Cogionis B. (2000). Nucleated RBCs—Significance in the peripheral blood film. Lab. Med..

[107] Schaefer M., Rowan R. (2000). The clinical relevance of nucleated red blood cell counts. Sysmex J. Int..

[108] Hajduk P., Copland M.D., Schultz D.A. (1992). Effects of capture on hematological values and plasma cortisol levels of free-range koalas (*Phascolarctos cinereus*). J. Wildl. Dis..

[109] Bolliger A., Backhouse T. (1960). The blood of the koala (*Phascolarctos cinereus*). Aust. J. Zool..

[110] Spencer A., Canfield P. (1995). Bone marrow examination in the Koala (*Phascolarctos cinereus*). Comp. Haematol. Int..

[111] Spencer A., Canfield P. (1994). Enhanced Heinz body formation, cell lysis and anaemia in a koala (*Phascolarctos cinereus*). Comp. Haematol. Int..

[112] Fabijan J., Sarker N., Speight N., Owen H., Meers J., Simmons G., Seddon J., Emes R., Tarlinton R., Hemmatzadeh F. (2020). Pathological findings in koala retrovirus-positive koalas (*Phascolarctos cinereus*) from Northern and Southern Australia. J. Comp. Pathol..

