# Supplementary material for: Health Assessments of Koalas after Wildfire: A Temporal Comparison of Rehabilitated and Non-Rescued Resident Individuals
Source: Animals (Basel). 2023 Sep 9;13(18):2863. doi: 10.3390/ani13182863 (PMC10525633; doi:10.3390/ani13182863)
Supplement: Supplementary file 1 [file animals-13-02863-s001.zip › animals-2574181-supplementary.pdf]

Supplementary material for Health assessments of koalas after wildfire: A temporal comparison of rehabilitated and non-rescued resident individuals

Table S1: list of all koalas including group, health check, sex, tooth wear class, presence of pouch young and outcome.

		Health checks						
**Koala name**	**Group**	**Rescue**	**Pre-release**	**Recapture**	**Sex**	**Tooth wear class**	**Presence of back(B)/pouch (P) young**	**Outcome**
Sterling	Rehabilitated	Yes			F	4		Died
Miles	Rehabilitated	Yes			M	4A	NA	Died
Allison	Rehabilitated	Yes			F	4	B	Euthanised
AJ	Rehabilitated	Yes			F	3	B	Euthanised
Billie	Rehabilitated	Yes			F	3		Euthanised
Isabella	Rehabilitated	Yes			F	2		Euthanised
Malu	Rehabilitated	Yes			M	5	NA	Euthanised
Ash	Rehabilitated	Yes			F	3	P	Released
Mallee	Rehabilitated	Yes			F	2	B	Released
Kim	Rehabilitated	Yes			F	3	P + B	Released
Hayley	Rehabilitated	Yes			F	1	NA	Released
Imogen	Rehabilitated	Yes			F	1	NA	Released
Kath	Rehabilitated	Yes			F	1	NA	Released
Camo	Rehabilitated	Yes			F	1	NA	Released
Montanna	Rehabilitated	Yes			F	2		Released
Gabou	Rehabilitated	Yes			M	5	NA	Released
Craig	Rehabilitated	Yes			M	4	NA	Released

Koala name	Group	Health checks<br>Rescue	Pre-release	Recapture	Sex	Tooth wear class	Presence of back(B)/pouch (P) young	Outcome
Beck	Rehabilitated	Yes			M	4	NA	Released
William	Rehabilitated	Yes			M	1	NA	Released
Tahlia	Rehabilitated	Yes	Yes		F	2	P	Released and GPS-tracked
Amelia	Rehabilitated	Yes	Yes		F	2	NA	Released and GPS-tracked
Jessie	Rehabilitated	Yes	Yes		F	3	P + B	Released and GPS-tracked
Tallow	Rehabilitated	Yes	Yes	Yes	M	2	NA	Released and GPS-tracked
Bear	Rehabilitated	Yes	Yes	Yes	M	3.5	NA	Released and GPS-tracked
Paul	Rehabilitated	Yes	Yes		M	3	NA	Released and GPS-tracked
Rick	Rehabilitated	Yes	Yes	Yes	M	5	NA	Released and GPS-tracked
Ian	Rehabilitated	Yes	Yes		M	4	NA	Released and GPS-tracked
Jarrah	Rehabilitated	Yes	Yes		M	2	NA	Released and GPS-tracked
Matthew	Rehabilitated	Yes	Yes		M	2.5	NA	Released and GPS-tracked
Mark	Rehabilitated	Yes	Yes		M	2	NA	Released and GPS-tracked
Hamish	Rehabilitated	Yes	Yes	Yes	M	2	NA	Released and GPS-tracked
Claire	Residents in burnt		Yes	Yes	F	4	P	GPS-tracked
Romane	Residents in burnt		Yes	Yes	F	4		GPS-tracked
Bronwen	Residents in burnt		Yes	Yes	F	3	P	GPS-tracked
Sage	Residents in burnt		Yes	Yes	F	4B		GPS-tracked
Peggy	Residents in burnt		Yes	Yes	F	2		GPS-tracked
Evan	Residents in burnt		Yes	Yes	M	4B	NA	GPS-tracked
Drew	Residents in burnt		Yes	Yes	M	5	NA	GPS-tracked
Jett	Residents in burnt		Yes		M	1	NA	GPS-tracked
Owen	Residents in burnt		Yes	Yes	M	2	NA	GPS-tracked

			Health checks					
**Koala name**	**Group**	**Rescue**	**Pre-release**	**Recapture**	**Sex**	**Tooth wear class**	**Presence of back(B)/pouch (P) young**	**Outcome**
Koala	Residents in unburnt		Yes		F	5	N	Euthanised
Rosalie	Residents in unburnt		Yes	Yes	F	3		GPS-tracked
Tamara	Residents in unburnt		Yes		F	4	P	GPS-tracked
Grace	Residents in unburnt		Yes		F	5	P	GPS-tracked
Annie	Residents in unburnt		Yes		F	5		GPS-tracked
Madonna	Residents in unburnt		Yes	Yes	F	4C	P	GPS-tracked
Mia	Residents in unburnt		Yes		F	1	NA	GPS-tracked
Zinger	Residents in unburnt		Yes		F	1	NA	GPS-tracked
Jaime	Residents in unburnt		Yes		F	2	P	GPS-tracked
Brandy	Residents in unburnt		Yes		F	2	NA	GPS-tracked
CS	Residents in unburnt		Yes	Yes	F	2		GPS-tracked
Xavier	Residents in unburnt		Yes	Yes	M	4A	NA	GPS-tracked
Rod	Residents in unburnt		Yes		M	5	NA	GPS-tracked
Murray	Residents in unburnt		Yes	Yes	M	4B	NA	GPS-tracked
Kenny	Residents in unburnt		Yes	Yes	M	5	NA	GPS-tracked
Alex	Residents in unburnt		Yes		M	2	NA	GPS-tracked
